# Lomitapide–a Microsomal Triglyceride Transfer Protein Inhibitor for Homozygous Familial Hypercholesterolemia

**DOI:** 10.1007/s11883-020-00858-4

**Published:** 2020-06-18

**Authors:** Claudia Stefanutti

**Affiliations:** Extracorporeal Therapeutic Techniques Unit, Lipid Clinic and Atherosclerosis Prevention Centre, Regional Centre (Lazio) for Rare Diseases, Immunohematology and Transfusion Medicine, Department of Molecular Medicine, “Sapienza” University of Rome, “Umberto I” Hospital, Rome, Italy

**Keywords:** Homozygous familial hypercholesterolemia, Lomitapide, Cardiovascular disease, Lipoprotein apheresis, Drug therapy

## Abstract

**Purpose of Review:**

Homozygous familial hypercholesterolemia (HoFH) is a rare, genetic condition characterized by high levels of Low density lipoprotein cholesterol (LDL-C); overt, early-onset atherosclerotic cardiovascular disease (ASCVD); and premature cardiovascular events and mortality. Lomitapide is a first-in-class microsomal triglyceride transfer protein inhibitor for the treatment of HoFH. This review provides an update on data emerging from real-world studies of lomitapide following on from its pivotal phase 3 clinical trial in HoFH.

**Recent Findings:**

Recent registry data have confirmed that HoFH is characterized by delayed diagnosis, with many patients not receiving effective therapy until they are approaching the age when major adverse cardiovascular events may occur. Data from case series of varying sizes, and from a 163-patient registry of HoFH patients receiving lomitapide, have demonstrated that lomitapide doses are lower and adverse events less severe than in the phase 3 study. Lomitapide enables many patients to reach European Atherosclerosis Society LDL-C targets. Some patients are able to reduce frequency of lipoprotein apheresis or, in some cases, stop the procedure altogether—unless there is significant elevation of lipoprotein (a). Modelling analyses based on historical and clinical trial data indicate that lomitapide has the potential to improve cardiovascular outcomes and survival in HoFH.

**Summary:**

Real-world clinical experience with lomitapide has shown the drug to be effective with manageable, less marked adverse events than in formal clinical studies. Event modelling data suggest a survival benefit with lomitapide in HoFH.

## Homozygous Familial Hypercholesterolemia

Homozygous familial hypercholesterolemia (HoFH) is a rare, genetic disorder of lipid metabolism that results in high LDL-C levels with or without concomitant elevation in lipoprotein (a) (Lp(a)) at a very early age [1, 2]. Patients with HoFH have extremely high LDL-C levels from birth, and this is associated with early-onset atherosclerotic cardiovascular disease (ASCVD) and aortic/supra-aortic valve disease. This, in turn, causes a range of associated life-threatening cardiac conditions [1]. If left untreated, HoFH is associated with ASCVD at a mean age of 12.5 years, with mean survival just 18 years [3].

The prevalence of HoFH is estimated to be 1:300,000 people [4], with founder effects evident in some populations with low levels of genetic admixture or high incidence of consanguineous marriages [5, 6]. The condition arises when an individual inherits two mutations that encode defects in proteins involved in the catabolism of LDL-C. Affected genes can include *LDLR*, *LDLRAP1*, *APOB*, and *PCSK9* [1]. The pattern of mutations is extremely varied, whereby patients may have a pair of matching mutations (“true” or “simple” homozygotes), a pair of unmatched mutations in the same allele (compound heterozygotes), or two unmatched mutations in two different alleles (double heterozygotes) [1]. Within these mutation patterns, there is further variability. LDL receptor (LDL-R), LDL receptor adapter protein 1 (LDLRAP1), and apolipoprotein B (ApoB) are responsible for clearing LDL-C from plasma, while proprotein convertase subtilisin/kexin type 9 (PCSK9) is responsible for inhibiting the recycling LDL-R to the cell surface by targeting the LDL-R for lysosomal degradation. Therefore, LDL-C levels will be elevated by loss-of-function mutations in *LDLR*, *LDLRAP1*, and *APOB* and by gain-of-function mutations in *PCSK9* [1]. The severity of the mutations results in wide phenotypic variability, whereby affected proteins can be completely or partially disabled [1].

### Long-Term Risks of HoFH—Data from Registries

Long-term analysis of groups of patients with HoFH has been conducted in the HoFH International Clinical Collaborators (HICC) registry, the Turkish A-HIT 1 registry, and the Lomitapide Observational Worldwide Evaluation Registry (LOWER). The 2018 data from the HICC registry has found that among 220 patients analyzed, mean (± SD) age at first myocardial infarction or aortic valve replacement was 35.7 ± 8.3 years, with patients as young as 17 years experiencing these events. Mean age of death was 42.4 ± 19.5 years (lower limit 13 years) most deaths (67%) were of cardiovascular origin [7•]. The Turkish A-HIT 1 registry of HoFH patents undergoing lipoprotein apheresis (LA) to control their condition evaluated 88 patients and found that mean age of diagnosis was delayed to 12 ± 11 years [8•], which is only 5 years before the HICC registry suggests that serious cardiovascular (CV) events may occur [7•]. These registry data serve to illustrate the need to diagnose HoFH early in the life of the patient and to apply effective treatments with the greatest urgency. Individual CV profiles ought to be carefully assessed in all patients regardless of age.

## Standards of Care

HoFH is extremely difficult to treat and requires inventive therapeutic solutions. For patients with common, polygenic causes of elevated cholesterol, the standard of care for lipid lowering is statins with or without ezetimibe. Statins work by inhibiting β-hydroxy β-methylglutaryl-CoA (HMG CoA), and one of the chief results of this is the increased expression of LDL-R via sterol regulatory element-binding protein (SREBPs [9]). This activity requires that the LDL-R is functional, even if only partially.

In patients with HoFH with little residual LDL-R activity (e.g., < 2%), statins are only able to reduce LDL-C levels by a modest amount (10–25%), even with maximal doses [1, 3, 10]. Other patients with less severe mutations may respond in part to statins.

For many years, a mainstay of therapy in HoFH has been LA. LA involves the extracorporeal removal of cholesterol and can reduce LDL-C levels by more than 50% and delay and interrupt the onset of ASCVD [11–13]. However, LDL-C levels rebound to baseline within 2 weeks of an LA procedure [14]. This means that patients using LA to control LDL-C levels require frequent sessions to avoid extended periods of elevated LDL-C levels, which exposes them to long-term risks of early-onset ASCVD [15]. For many patients, LA needs to be applied weekly to maintain interval LDL-C levels below European Atherosclerosis Society (EAS) targets. However, LA is undoubtedly a burden on time, education, and work. Attendance at treatment centers can be problematic, and LA is not universally available in all countries and regions [16, 17].

On the other hand, LA has been demonstrated to improve life expectancy and delay morbidity associated with ASCVD. This was shown in the recent Sino-Roman Study in which Italian HoFH patients receiving lipid-lowering therapies (LLTs), including lomitapide, plus LA had significantly greater survival and less incidence of (CVD) compared with Chinese HoFH patients who received standard LLTs but neither LA nor lomitapide (Table 1) [18••]. Importantly, LA has the capability to reduce levels of Lp(a) [19], and the Sino-Roman study showed that elevated levels of Lp(a) is associated with increased mortality in HoFH [18••]. Therefore, LA has the potential to improve survival via removal of lipid species that are refractory to pharmacological therapy. LA also has a range of pleiotropic benefits including downregulation of pro-inflammatory mediators, possibly via alterations in transcription of cytokine mRNA [19]. Moreover, LA induces hemodynamic and hemorheological effects at any arterial district, including coronary arteries, thereby improving myocardial perfusion [20].

**Table 1:** Lipid-lowering treatments and outcomes of the homozygous FH patients from the Italian and Chinese centers [18]

Variable	Italy (*n* = 18)	China (*n* = 44)	*P* value
Age started pharmacotherapy treatment (years)	5.6 ± 3.4	10.7 6 4.6	< 0.001
Lipid-lowering drugs (%)	94.4	95.5	0.866
Statins (%)	88.9	95.5	0.339
Ezetimibe (%)	77.8	81.8	0.715
Resins (%)	66.7	0	< 0.001
Fibrates (%)	16.7	0	0.022
Probucol (%)	0	77.3	< 0.001
PCSK9 inhibitors (%)	0	0	–
Lomitapide (%)	61.1	0	< 0.001
Age started lomitapide (years)	23.5 ± 4.1	–	–
Lipoprotein apheresis (%)	100	0	< 0.001
Age started apheresis (years)	8.9 ± 5.5	–	–
Treated LDL cholesterol (mmol/L)	6.6 ± 2.7*	13.1 ± 2.7	< 0.001
ΔLDL cholesterol (mmol/L)	12.5 ± 5.0	2.6 ± 3.6	< 0.001
Treatment duration (years)	17.4 ± 8.8	5.5 ± 4.8	< 0.001
LDL cholesterol life (years)^†^	258.9 ± 98.7	227.3 ± 112.8	0.305 0.792^‡^
Mean LDL cholesterol exposure/year (mmol/L)^†^	12.4 ± 3.0	14.6 ± 3.0	0.011 < 0.001^‡^
CVD event, *n* (%)	4 (22.2)	20 (45.5)	0.088
Age at CVD event (years)	19.0 ± 9.6	16.1 ± 5.8	0.421
Death, *n* (%)	3 (16.7)	14 (31.8)	0.225
Age at death (years)	20.3 ± 10.7	17.9 ± 6.2	0.586

Continuous variables are expressed as mean 6 standard deviation, and categorical variables are expressed as proportions (Permission will be needed from Elsevier to reproduce this.)

*CVD* cardiovascular disease, *LDL* low-density lipoprotein, *PCSK9* proprotein convertase subtilisin/kexin type 9

*Time-average LDL cholesterol calculated using Kroon’s equation [14]

†Censored at age 30 years

^‡^Adjusted for (baseline) untreated LDL cholesterol

In common with statins, the recently developed PCSK9 inhibitors, which work by enhancing the cellular recycling of LDL-R, also require residual LDL-R function to reduce plasma LDL-C levels [21]. The difficulties in using PCSK9 inhibitors in all patients with HoFH were borne out by results from the TESLA and TAUSSIG trials, which demonstrated that the PCSK9 inhibitor evolocumab had diminished activity in HoFH patients with negative functional mutations versus those with mutations allowing some residual LDL-R activity [22, 23]. Among all the HoFH patients in the TESLA trial, response was highly variable, whereby evolocumab reduced LDL-C by approximately 20%, but with a standard deviation of 24% [22]. In the TAUSSIG trial, responses were similarly variable and an analysis by mutation type revealed consistently poor responses in HoFH patients with null *LDLR* mutations [23]. Long-term data from TAUSSIG suggests a mean response rate of evolocumab in FH of 21.2% [24]. The issues with variability of response to PCSK9 inhibitors in HoFH indicate a need to carefully select therapies based on available genetic profiling [25]. Expert opinion underscores the limitations of PCSK9 inhibitors in HoFH and suggests that they may not have the broad applicability required to reduce the burden of LA, certainly for HoFH patients with receptor-negative mutations [26].

Given the problems associated with statin- and PCSK9-based therapy in HoFH, other treatment modalities have been sought, including an anti-sense oligonucleotide that targets mRNA for ApoB [27] (now discontinued) and lomitapide.

## Lomitapide

Lomitapide is an oral microsomal triglyceride transfer protein inhibitor that prevents assembly of apoB-containing lipoproteins in the liver and intestines [28]. The drug works by binding directly to microsomal triglyceride transfer protein (MTP) in the endoplasmic reticulum of hepatocytes and enterocytes, which inhibits the assembly of VLDL and chylomicrons [28, 29]. MTP is responsible for the modulation of the number of ApoB-containing particles that are released into the blood, and interestingly, rare, recessive mutations in MTP lead to abetalipoproteinemia, which is characterized by ultralow levels of plasma cholesterol and complete absence of plasma LDL-C and ApoB [29, 30].

Lomitapide is licensed as an adjunct to a low-fat diet (< 20% energy from fat) and other lipid-lowering medicinal products with or without LA in adult patients with HoFH. The introduction of lomitapide has provided an additional option to tailor LLT in HoFH to deliver the therapeutic intensity required in HoFH to mitigate the risk of ASCVD. Patients receiving lomitapide should take daily oral dietary supplements that provide 400 IU vitamin E and approximately 200 mg linoleic acid, 110 mg eicosapentaenoic acid, 210 s mg alpha linolenic acid, and 80 mg docosahexaenoic acid per day.

### Clinical Trials

In an open-label phase 3 trial of lomitapide in adult patients with HoFH (NCT00730236), lomitapide was dosed according to an escalation to maximum-tolerated dose. All patients were advised to follow a diet in which < 20% of energy was derived from fat. Background LLTs were maintained, and this included LA for 18 of the 29 enrolled individuals. Over 26 weeks, lomitapide resulted in median reductions in plasma LDL-C of 50%. ApoB levels were reduced by 49% [31]. These results were largely maintained out to the planned 78 weeks of the pivotal trial. LDL-C levels in the 23 patients remaining at week 78 were 38% lower than baseline [31]. Given the mode of action of lomitapide, it was not surprising that the most predominant adverse events were gastrointestinal in nature. Elevations in liver function test (LFTs) > 5× ULN occurred in four patients, but these resolved with temporarily interruption of lomitapide [31]. On the basis of the results of this study, lomitapide was licensed for use as an adjunct to low-fat diet in adult patients with HoFH in the USA and Europe and areas of North America, Latin America, and Asia.

Patients who completed the 78-week pivotal phase 3 study were eligible to enroll in an extension phase (NCT00943306) in which lomitapide was administered for a total of 294 weeks. Nineteen patients enrolled in this phase [32]. At week 246 of the extension phase of the phase 3 trial, 14 (74%) and 11 (58%) of the 19 remaining patients reached LDL-C targets of 100 mg/dL and 70 mg/dL, respectively, at least once during the study period [32]. The extension phase revealed information about the long-term adverse event profile of lomitapide. In common with the phase 3 study, most adverse events were gastrointestinal, commensurate with the mode of action of the drug. The most frequently reported events were diarrhea, nausea, dyspepsia, and vomiting [32]. Twenty-one percent of patients experienced liver function test (LFT) excursions ≥ 5× upper limit of normal, but these were usually were associated with concomitant use of cytochrome P450 3A4 inhibitors or excess alcohol. In all cases, elevated LFTs were managed with a temporary dose decrease or interruption in lomitapide [32].

Both the phase 3 trial and the extension phase revealed elevations in hepatic fat in patients taking lomitapide; however, this has not been associated with elevated LFTs in all patients, and no overt liver disease has been attributable to lomitapide therapy. Nevertheless, lomitapide has been subject to a Risk Management Plan (RMP) to monitor long-term effects of lomitapide on the liver [32]. In common with general findings that adverse events to lomitapide regress over time, the incidence of drug-related adverse events in the extension phase of the lomitapide phase 3 trial was lower than that in the earlier core study (84.2% versus 42.1%) [32].

A further phase 3 study was conducted in Japan (NCT02173158) in which nine adult HoFH patients were administered lomitapide over the course of a core 26-week period and a subsequent 30-week follow-up [33]. LLT and dose escalation were conducted according to the phase 3 pivotal trial. Reductions in mean LDL-C levels were similar to those in the pivotal phase 3 trial (42%). In common with the phase 3 trial, some patients were able to increase their LA intervals while maintaining reductions in plasma LDL-C. Six patients were able to achieve LDL-C < 100 mg/dL at least once during the efficacy phase [33]. Adverse events mirrored those of the pivotal trial [33]. The Japanese trial also had an extension phase that enrolled five of the eight patients who completed the core study [34]. A mean 35.6% reduction in LDL-C levels was observed between the end of the 26-week efficacy phase of the core study and 60 weeks of follow-up in the extension study. Two of the patients achieved LDL-C levels < 100 mg/dL. Adverse events were similar to the core study [34].

The overall picture from the clinical trials of lomitapide is that the drug is effective in lowering LDL-C and that many patients can achieve levels < 100 mg/dL. This occurs regardless of the application of LA, and LA intervals can be extended in patients receiving the drug—however, it should be borne in mind that LA has other effects such as reduction in Lp(a) levels and modulation of inflammatory mediators [35] that have not been recorded for lomitapide. Adverse events associated with lomitapide are chiefly gastrointestinal and relatively straightforward to manage.

### Real-World Evidence

In rare diseases, such as HoFH, there is an ongoing problem of data availability and clinical trial size. In contrast with other, more common diseases, clinical trials of orphan drugs in rare disease are smaller due to the size of the available pool of patients. Additionally, once a drug for a rare disease is in general clinical use, large bodies of “real-world” evidence (RWE) or “big data,” as it is often known, will never become available. This makes it extremely difficult to conduct analyses such as median time of survival.

In addition to the limitations of formal data availability, for lomitapide, there is an additional technical aspect of the clinical trial that means that the drug is used differently in the clinic than in the phase study. In the phase 3 study, lomitapide was dosed according to a maximum-tolerated dosing regimen [31]. In this schema, patients undergo dose escalation until an adverse event occurs that requires dose titration. In contrast, in the clinic, patients undergo dose escalation only until desired efficacy is reached. For lomitapide, this means that the doses seen in reports of real-world use of the drug are lower than that in the clinical trial [36]. Lower doses have the potential to drive down the incidence of adverse events.

**Fig. 1:**
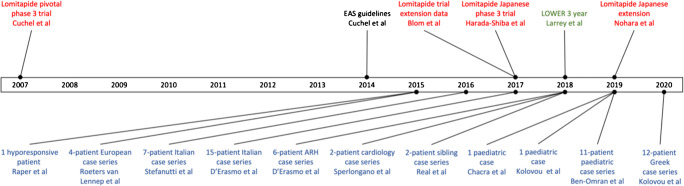
Data sources for lomitapide from clinical trials (red), registries (green), and case reports (blue)

RWE on lomitapide has become available both from patient case reports and from registry data (Fig. 1). One of the largest case series evaluated outside of a clinical trial setting was that of an Italian cohort of 15 adults with HoFH. Importantly, ten of the patients had mutations in *LDLR*, and five had autosomal recessive hypercholesterolemia (ARH) [36]. ARH is a rare autosomal recessive form of HoFH. It is caused by mutations in *LDLRAP1*, which codes for the low-density lipoprotein receptor adaptor protein-1. This protein plays a key role in the internalization of the LDL-R/LDL-C receptor complex, and loss-of-function mutations in the gene result in LDL-C remaining in circulation rather than being internalized by hepatocytes [37–40].

In the Italian study, HoFH patients, including five with ARH, were treated with lomitapide for more than 6 months, along with standard LLTs. LA was conducted in 10 of the patients. Mean LDL-C levels decreased by 68%, and this was achieved with a mean lomitapide dose of 19 mg/day [36]. This contrasts sharply with the median dose of 40 mg/day in the pivotal phase 3 trial [31]. On this lower dose, none of the patients discontinued lomitapide, and there were no problematic elevations in LFTs [36]. A subset of patients were monitored with liver ultrasound (*n* = 5) or magnetic reasonance imaging (MRI) (*n* = 1). However, this was not conducted in a comprehensive manner, so conclusions on hepatic steatosis cannot be reliably determined [36].

A follow-up study was conducted by the same group in Italy, whereby electronic case reports from 52 patients with ARH were examined. Mean follow-up was 14.1 ± 7.3 years. Among the patients, high doses of statins and ezetimibe were recorded, and just over half of the patients were receiving apheresis. Six of the patients (11.5%) had received lomitapide [37]. Compared with other lipid lowering medication, patients receiving lomitapide had the greatest decreases in LDL-C levels, despite two of the patients stopping LA. LDL-C levels were reduced by 88.3 ± 5.0 mg/dL compared with 62.0 ± 22.5% for statins plus ezetimibe and 70.6 ± 10.3% for the same regimen with LA added. The research group commented that the use of lomitapide in ARH warrants further attention [37].

Staying with Italy, Stefanutti et al. described seven cases of genetically determined HoFH treated with lomitapide in a general clinic setting in Rome [41]. Following dose titrations, lomitapide doses were in the range 10–30 mg/day for five of the patients. One patient received a higher dose at 60 mg/day, and another patient was on just 5 mg/day. Three of the patients achieved LDL-C reductions of > 50%. The patient on the very low lomitapide dose did not experience appreciable decreases in LDL-C. Gastrointestinal adverse events were managed by adjustments to intake of dietary fat [41]. An Italian cardiology unit also identified two HoFH cases by applying clinical criteria followed by genetic confirmation of homozygosity [42]. One of these patients achieved a 78% reduction in LDL-C to 66 mg/dL on lomitapide 20 mg/day. The other patient achieved a 60% reduction to 107 mg/dL on just 5 mg/day with background statins and ezetimibe [42]. In both patients, hepatic ultrasound showed healthy livers without steatosis or fibrosis [42].

**Fig. 2:**
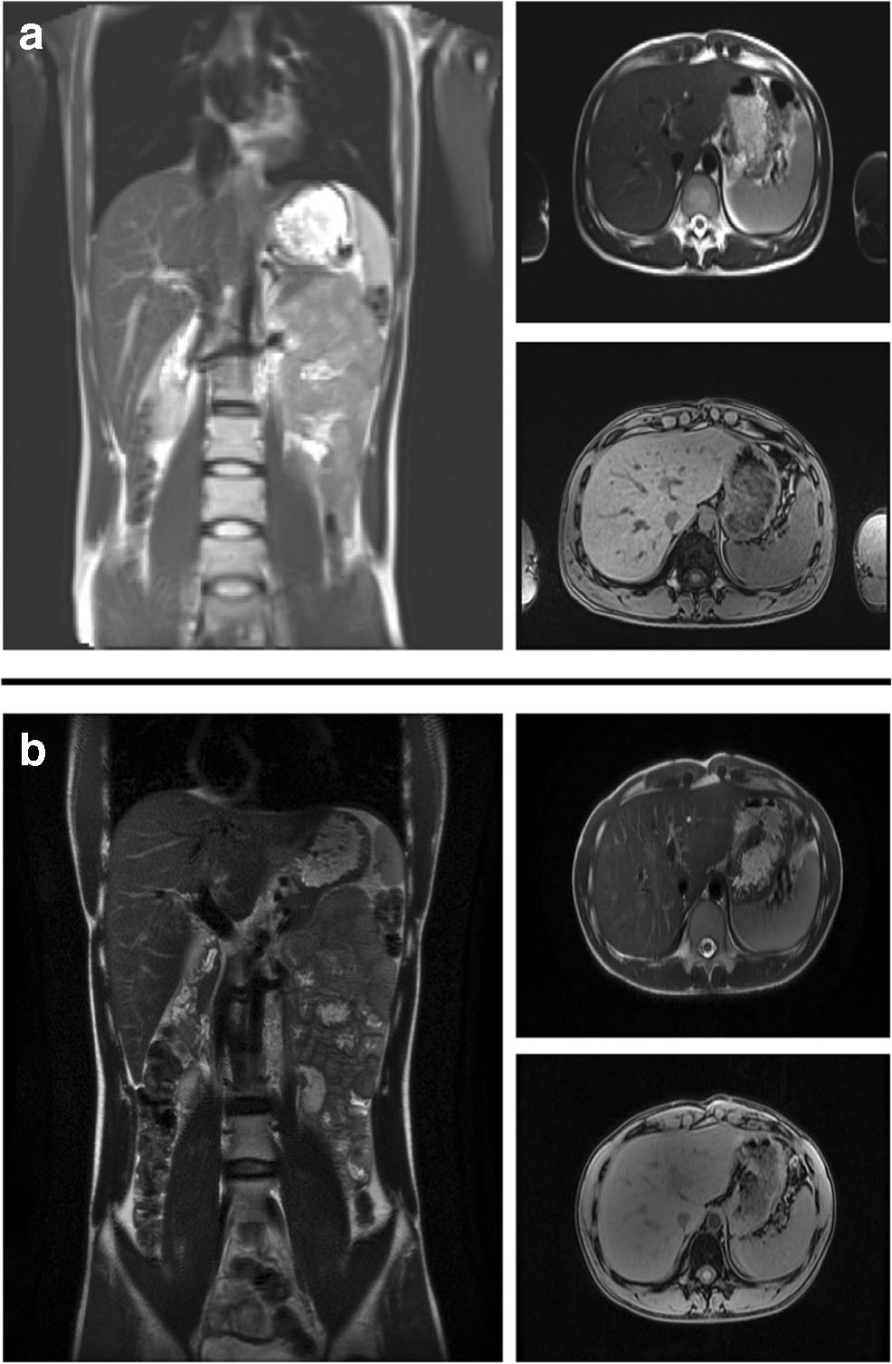
Hepatic MRI for patient 3 in **a** 2015 and **b** 2018 (unpublished data)

**Fig. 3:**
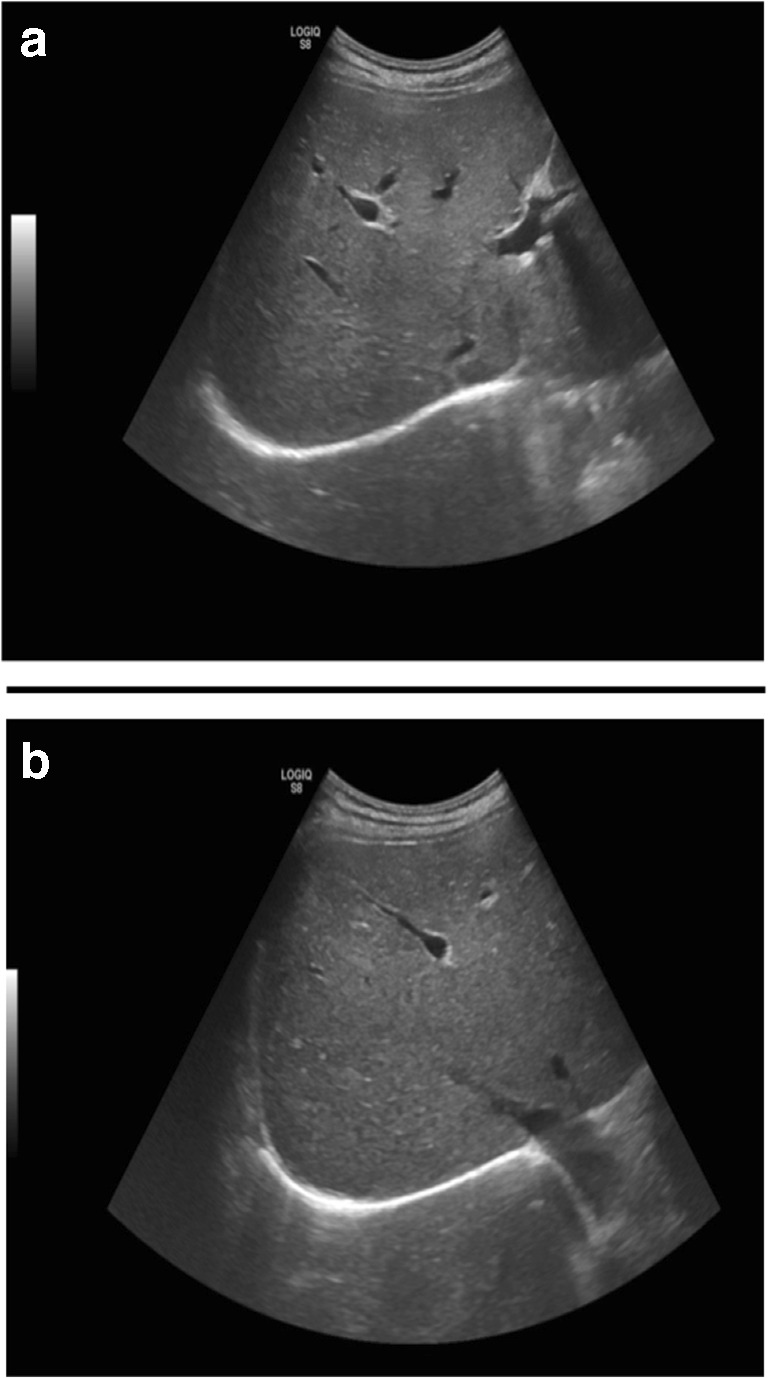
Hepatic ultrasound for patient 5 in **a** 2014 and **b** 2019 (unpublished data)

While hepatic steatosis is also common with lomitapide, and the long-term implications of this remain unclear, there are follow-up data from the Roman Stefanutti group that indicate that steatosis can persist in patients receiving the drug. In the Roman cohort, steatosis was determined with a combination of ultrasound and MRI. In two patients, diffuse and/or slight hepatic steatosis was evident in up to 8 years of follow-up (Table 2). MRI from patient 3 is pictured in Fig. 2 and shows no evidence of steatosis at follow-up. In contrast, the ultrasound from patient 4 (this patient could not have an MRI due to a replacement aortic valve.) shows evidence of slight steatosis 3 years after commencement of lomitapide (Fig. 3). Slight steatosis that was evident in patient 2 also appears to have persisted, but the imaging technique for this patient was changed between 2016 and 2019. In the Rome clinic, patients are routinely followed up with MRI and ultrasound depending on the evolution of liver abnormalities in patients receiving lomitapide as this is considered to be important while we continue to gather information on the long-term impact of the drug on the liver.

**Table 2:** Hepatic steatosis data from 9 HoFH patients treated with lomitapide in Rome (unpublished data)

Pt	Lomitapide Start, year dose	LA start, year	Baseline liver US, year	Baseline steatosis	Last US, year	Steatosis on last US	Baseline MRI, year	Baseline Steatosis on MRI	Last MRI, year	Steatosis on last MRI
1	2010 5–60 mg/day	1990	NA	NA	NA	NA	2010	None	2018	Diffuse
2	2015 5–30 mg/day	1996	2015	Slight	2019	Slight	2016	Slight	NA	NA
3	2014 5–30 mg/day	2003	2015	None	2019	None	2016	Slight	2019	None
4	2015 5–25 mg/day	2004	2015	None	2018	Slight	2015	None	2018	Slight
5	2014 5–30 mg/day	1996	2014	None	2019	None	N/A	N/A	N/A	N/A
6	2014* 5–20 mg/day	1993	2015	None	Nov 2017 (regression)	None	2016 May	Diffuse	N/A	N/A
7	2015* 5–15 mg/day	2011	2015	None	2016	None	NA	N/A	N/A	N/A
8	2019* 5 mg/day	1989	2010	None	August 2019	Mild	2019 April	None	N/A	N/A
9	2019 5 mg/day	2000	January 2019	None	October 2019	Slight	N/A	N/A	N/A	N/A

*LA* lipoprotein apheresis, *MRI* magnetic resonance imaging, *N/A* not applicable, *US* ultrasound

*Lomitapide discontinued at date indicated

Elsewhere, a small number of case series of different sizes have been published. A 4-patient, multinational case series was published by Roeters van Lennep et al., who observed LDL-C reduction with lomitapide in the range 35–83%. The patients were receiving a range of background LLT, but one patients was treated with lomitapide alone [43]. A larger patient series from a Greek group followed 12 HoFH patients for 3–24 months (median 13.8 ± 7.9) of lomitapide therapy [44]. The patients were analyzed in two groups, one with standard LLT, and the other with LLT plus LA. Over and above reductions in LDL-C levels achieved with background LLT; lomitapide reduced LDL-C levels by 56.8% and 54% in these groups, respectively, at an average dose of 21.4 ± 10.5 mg/day [44].

An interesting case history has been recently published of two Spanish brothers with HoFH and poor response to standard LLTs [45•]. One of the brothers had renal failure (two transplants), and a range of concomitant medications would have made him ineligible for a clinical trial. The patient had also undergone a triple coronary bypass. The other brother was more straightforward but had also undergone a triple bypass. Both brothers were receiving apheresis at the time lomitapide was initiated. LDL-C levels reduced to < 100 mg/dL in both patients, and the first brother was able to stop LA [45•].

Not all patients can benefit from low doses of lomitapide. It is known that there can be variability in response to the drug [46], and it is possible that this is driven by phenotypic variability in MTP. This notion is borne out by data from a study of four HoFH cases from Greece treated with lomitapide in which two patients were characterized as “hyper-responders” (LDL-C reductions > 50%) and two as “hypo-responders” (LDL-C reductions < 50%). Genetic analysis revealed 36 different mutations in MTP, among which the two hyper-responders shared six common gene variants. None of these variants was present in the hypo-responders [47]. An example of hypo-responsiveness was evident in a 2015 case from a Hispanic female [48]. Raper et al. continued to treat a HoFH patient who had been on the maximum 60 mg/day dose of lomitapide in the pivotal phase 3 trial. Attempts were made to reduce the dose to 40 mg/day, but the patient experienced rebound in LDL-C characteristic of hypo-responsiveness. However, the success in this patient lies in that she was able to achieve LDL-C levels of just 50 mg/dL with discontinuation of LA and only a transient increase in LFTs at the time of the re-escalation in lomitapide dose [48].

Despite the need to diagnose and treat HoFH as early as possible in life, at the time of writing, lomitapide does not have a license in pediatric patients. However, a case series has been published in which 11 HoFH patients all < 18 years old (mean age 11.6 ± 1.1 years) were treated with lomitapide in accordance with the adult label, albeit with smaller starting doses (2.5 mg/day) [16]. The patients were split between 10 centers in eight countries, so there was variability in practice patterns, availability of LA and reimbursement policies [16]. Addition of lomitapide to standard background LLT resulted in a mean decrease in LDL-C of 58.4 ± 6.8%. Six of the patients achieved EAS pediatric LDL-C target levels < 135 mg/dL. Among the six patients who were receiving LA, all of them had their LA frequency reduced, and three were able to stop LA altogether. One patient, who was on a twice-weekly LA schedule, was able to reduce his LA burden by 75%. Adverse events were similar to other studies with a predomination of manageable GI disturbances and transient LLT excursions [16].

An earlier single-patient case study was reported of a 7-year-old girl with HoFH who was treated with lomitapide for 4 years [49]. The care team used the same principles of therapy as for adult patients (fat restriction, background statins and essential fatty acids, and vitamin E supplementation). The patient achieved a 37% reduction in LDL-C down to a nadir of 232 mg/dL with lomitapide 20 mg/day and no LA [49].

In Greece, an 8-year-old boy with HoFH and extensive xanthomas, with systolic murmur in both carotid arteries and aortic valve stenosis, was treated unsuccessfully with statins, in a setting where LA was not an option due to issues of both venous access and parental consent [50]. LDL-C levels were very high at 1050 mg/dL, and the statin regimen was only able to get this down to 866 mg/dL. Treatment with lomitapide escalated to 40 mg/day resulted in LDL-C levels reaching 390 mg/dL. There was notable regression of xanthomas, and no side effects were reported [50]. In this case, lomitapide resulted in considerable LDL-C reduction, but still insufficient to protect the patient against long-term evolution of atherosclerosis and related cardiovascular complications, and the patient remained on combination LLT therapy.

LOWER is beginning to yield evaluable data on the long-term effects of lomitapide in a large number of patients. As of March 2017, 163 patients were enrolled in the registry, and Larrey et al. conducted an analysis of longitudinal liver safety with 47.1 months mean exposure to lomitapide [51••]. Mean lomitapide dose was 10 mg/day. Larrey et al. confirmed that LFT excursions are common in patients receiving lomitapide but that the magnitude of these is a lot less than in the phase 3 trial, with only 6.3% of patients experiencing transaminase levels > 5× upper limit of normal over 3 years in LOWER versus 9% in the 28-week clinical trial [51••]. Hepatic abnormalities have been recorded for approximately 15% of patients in the registry. The use of imaging to determine hepatic steatosis/fibrosis is patchy but will be available from the European cohort because imaging or biomarker-based assessment of hepatic parameters is mandatory in Europe [52]. No patient in LOWER has satisfied the criteria for Hy’s Law [51••].

The totality of the currently available RWE on lomitapide strongly indicates when the drug is used without the forced maximum tolerated dose (MTD) titration that characterized the pivotal phase 3 trial; most patients and care teams find that the drug can be used at lower doses than the median trial dose and therefore with fewer side effects. Lomitapide can allow for increases in LA intervals and, in a minority of patients, even cessation of the procedure, providing that mean interval LDL-C and Lp(a) levels are under control and there is no evidence of progression of ASCVD.

### CV Outcomes for Lomitapide

As described above, there are insufficient patients with HoFH to generate data on cardiovascular event rates and survival. Nonetheless, researchers have made attempts to estimate the possible benefits of lomitapide on CV events by conducting modelling analyses based on existing data. In a modelling analysis by Leipold et al., an assumption was made on the basis of the phase 3 pivotal trial of lomitapide in HoFH that the drug was capable of eliciting a conservative 38% reduction in LDL-C levels [53]. Using an historical study of mortality reduction in a cohort of 149 South African HoFH patients [54], it was possible to calculate age-dependent hazards and treatment-dependent hazard ratios for mortality and time to first major adverse cardiovascular events (MACE). It was estimated that for every millimoles per liter of LDL-C reduction, the relative risk of mortality decreased by 23%, and the risk of a major adverse cardiovascular event reduced by 15%. Importantly, using the same data, the mean life expectancy was estimated to increase by 5.7 years if lomitapide was commenced at age 18 years and by 6.7 years if the drug was commenced at birth [53].

A further modelling analysis has been conducted using data derived from the phase 3 pivotal trial of lomitapide. In this instance, CV event rates were estimated along with the proportion of patients achieving EAS guideline targets of ≤ 100 mg/dL and ≤ 70 mg/dL. These data were compared with additional data derived from clinical trials of evolocumab and mipomersen [55]. The lomitapide analysis used data from all 29 patients enrolled in the phase 3 study. Over the first 26 weeks of the phase 3 trials, 15 patients reached the ≤ 100 mg/dL LDL-C target at least once, and eight (28%) reached the ≤ 70 mg/dL target. In the extension study, 19 patient remained on lomitapide, and of these, 14 (74%) reached the ≤ 100 mg/dL LDL-C target at least once, and 11 (58%) reached the ≤ 70 mg/dL target [55]. Only two MACE were reported in the lomitapide trial, which equates to 1.7 events per 1000 patient months of treatment. Corresponding rates for mipomersen and evolocumab were 9.5 and 1.8, respectively. On-treatment LDL-C levels for lomitapide, mipomersen, and evolocumab were estimated to be 166, 331, and 286 mg/dL, respectively [55]. Additional data on CV endpoints may become available on evolocumab and lomitapide, but mipomersen has been discontinued by the manufacturer, and no further information will be available on this drug.

These results together with those of Leipold et al. support the notion that lomitapide has the potential to reduce CV event rates in the general population of HoFH patients receiving the drug, which may have important benefits in terms or reducing the burden of treating CV events.

## Conclusions

HoFH is a rare, genetic disorder of lipid metabolism that causes extremely elevated high LDL-C levels from birth, resulting in severe and aggressive CVD at a very early age. HoFH is an extremely difficult to treat disease that requires multiple therapeutic interventions in the pursuit of target LDL-C levels and to control levels of Lp(a). Recent registry data have become available from HICC and A-HIT which emphasize the ongoing high CV risk of HoFH and the impact of HoFH on Quality of life despite the availability of lipid lowering therapies such as statins and apheresis, highlighting the need for additional therapeutic options.

On the basis of a phase 3 clinical trial, lomitapide was licensed for use as an adjunct to low-fat diet and lipid lowering therapies, including apheresis, in adult patients with HoFH. Lomitapide has been available for long enough now for many physicians to have used it in patients outside of clinical trials. When the drug is used without the forced MTD titration in the phase 3, most care teams are finding that the drug can be used at lower doses than the median trial dose and therefore with fewer side effects. Despite the lower median dose, the LDL-C lowering effect is equivalent or greater than that observed in the clinical studies with the majority of patients achieving LDL-C target levels.

Lomitapide can enable adjustment of LA schedules to reduce therapeutic burden. In a minority of HoFH patients, a complete cessation of LA can be achieved, provided that target LDL-C (estimated by the calculation of mean LDL-C interval between LA sessions) is achieved and that cardiovascular imaging of coronary arteries and aortic valve is reported to be stable. However, the coexistence of elevated Lp(a) levels may contraindicate cessation of LA as elevated Lp(a) has been associated with increased mortality in HoFH [18••]. Careful serial monitoring of the liver by means of liver imaging is essential.

Recent modelling data demonstrate that, in the absence of outcomes data, there is a potential benefit of lomitapide in terms of life years gained, decrease in time to major adverse CV events, and EAS target attainment. This has the potential not only to improve survival but also to avoid treatment of the costly sequelae of clinically significant CVD.
